# Pattern classification of EEG signals reveals perceptual and attentional states

**DOI:** 10.1371/journal.pone.0176349

**Published:** 2017-04-26

**Authors:** Alexandra List, Monica D. Rosenberg, Aleksandra Sherman, Michael Esterman

**Affiliations:** 1 Department of Psychology and Neuroscience Program, Hamilton College, Clinton, New York, United States of America; 2 Department of Psychology, Yale University, New Haven, Connecticut, United States of America; 3 Department of Cognitive Science, Occidental College, Los Angeles, California, United States of America; 4 Research Service, VA Boston Healthcare System, Boston, Massachusetts, United States of America; 5 Department of Psychiatry, Boston University School of Medicine, Boston, Massachusetts, United States of America; University of British Columbia, CANADA

## Abstract

Pattern classification techniques have been widely used to differentiate neural activity associated with different perceptual, attentional, or other cognitive states, often using fMRI, but more recently with EEG as well. Although these methods have identified EEG patterns (i.e., scalp topographies of EEG signals occurring at certain latencies) that decode perceptual and attentional states on a trial-by-trial basis, they have yet to be applied to the spatial scope of attention toward global or local features of the display. Here, we initially used pattern classification to replicate and extend the findings that *perceptual* states could be reliably decoded from EEG. We found that visual perceptual states, including stimulus location and object category, could be decoded with high accuracy peaking between 125–250 ms, and that the discriminative spatiotemporal patterns mirrored and extended our (and other well-established) ERP results. Next, we used pattern classification to investigate whether spatiotemporal EEG signals could reliably predict *attentional* states, and particularly, the scope of attention. The EEG data were reliably differentiated for local versus global attention on a trial-by-trial basis, emerging as a specific spatiotemporal activation pattern over posterior electrode sites during the 250–750 ms interval after stimulus onset. In sum, we demonstrate that multivariate pattern analysis of EEG, which reveals unique spatiotemporal patterns of neural activity distinguishing between behavioral states, is a sensitive tool for characterizing the neural correlates of perception and attention.

## Introduction

Over the last decade, multivariate pattern-classification analyses of fMRI BOLD signals have emerged as a fruitful approach for using neural activity to decode various behavioral states including perceiving, attending to, and imagining features, objects, and scenes (for reviews, see [1–4]). Recently, pattern-classification analyses have also been applied to electroencephalography (EEG) signals (e.g., [5–16]). This application to EEG has extended the standard event-related potential (ERP) analyses in which a critical electrode (or a cluster of electrodes) is selected within a specific scalp region (based on data inspection and/or prior results), and the trial-averaged stimulus-evoked EEG signals (i.e., ERPs) from the selected electrode(s) are compared between conditions. Instead, as applied here, multivariate classification techniques can reveal, in an agnostic data-driven manner, topographic weightings of EEG signals that maximally distinguish specific perceptual, attentional, or behavioral states within a given time interval. Thus, pattern-classification analyses offer greater sensitivity than standard ERP analyses by simultaneously integrating information across electrodes. Because pattern-classification analyses identify EEG correlates with high sensitivity, they are typically evaluated by how well they predict the corresponding perceptual, attentional, or behavioral states on a trial-by-trial basis (rather than how well trial-averaged signals from selected electrodes differentiate experimental conditions, as in standard ERP analyses). Cross-validated predictive measures, like the ones we use here, are also less susceptible to false positives than analyses traditionally applied to ERPs, because inaccurate models will not generalize to the held-out data.

The first aim of the current study is to replicate and extend prior EEG applications of pattern-classification analyses toward decoding perceptual states. Although prior studies have applied similar analyses toward classifying object category (e.g., faces versus cars), they have done so in the context of challenging stimulus discriminations (using stimulus degradation or distraction [5–7, 10–13]). These previous studies were aimed at decoding individual differences in perception and decision-making, and used a variety of algorithms and feature-selection for classification. In contrast, in our first experiment, we examined passive viewing of clearly discernable stimuli using classification methods common in the fMRI literature (e.g., [17–19]), in order to determine the spatiotemporal profile underlying successful pattern-classification of relatively “simple” visual perception. This experiment further serves as a benchmark of our particular classification methods, and as a model system for comparing perceptual states in which known ERP markers exist.

Thus, in Experiment 1, we first examined EEG correlates for distinguishing object category (i.e., faces and non-face Gabors), as well as two extensions, face orientation (i.e., upright and inverted faces) and spatial position (i.e., left and right stimulus locations), for which prior studies using standard ERP analyses have shown robust differences over specific electrode sites (i.e., ERP components). Specifically, the N170 ERP distinguishes between seeing faces versus non-face objects [20–22] or seeing upright versus inverted faces (e.g., [23]). Similarly, both perceiving and attending to stimuli in the left versus right visual field can be distinguished on the basis of the contralateral posterior ERP components, such as the P1, N1, N2Pc and CDA/SPCN (e.g., [24–30]). Thus, a broad goal of the first experiment was to demonstrate the sensitivity of the pattern-classification technique in distinguishing perceptual features from single-trial EEG data that have well-established ERP markers, in the absence of stimulus degradation, distraction or challenging behavioral demands.

Despite the advances in using pattern-classification analyses to identify EEG correlates that are associated with stimulus categories, task difficulty, performance level, and attentional readiness (e.g., [5–7, 12–13]), less work has been done to explore the ability of pattern classification to decode subjective states of covert visuo-spatial attention. To our knowledge, few studies have conducted pattern-classification analyses of EEG for identifying distinct attentional states (e.g., [10, 14, 15, 31]; note that various others have focused on other EEG-derived signals, e.g., steady-state evoked potentials: [16]). Thiery and colleagues [14] were successful in decoding the locus of covert visual attention using ERP data from *a priori* defined temporal windows and spatial locations (i.e., electrodes). As previously stated, we instead wanted to apply pattern-classification analysis without such *a priori* assumptions on single-trial EEG data. Kasper and colleagues’ [10] and Treder and colleagues’ [15] classification procedures most closely approach ours in that respect. Kasper et al. [10] successfully isolated attentional successes versus failures in an attentional blink study: From EEG averaged over 20-ms time bins, they decoded the ability of perceivers to identify the (second) target that is susceptible to the attentional blink. Treder and colleagues [15] differentiated attended versus unattended *auditory* pattern deviants from EEG voltage data averaged over data-defined time windows, consistent with a P3 timecourse (the P3b ERP differentiates task-relevant deviant from repeated stimuli [32]). Like us, they also identified electrodes whose signals were most strongly differentiated between conditions, and they showed critical spatial topographies akin to those found for the P3 ERP. Treder et al.’s [15] findings are powerful in demonstrating the ability to use pattern classification to identify spatial topographies of covert auditory attention, for which a robust single-trial ERP is detectable. In Experiment 2, we complement and extend their results by examining single-trial EEG pattern classification for the scale of visual attention, for which, importantly, no consistent ERP differences are reported, and thus provides a viable alternative to standard ERP analyses.

Thus, the second aim of the current study is to apply pattern-classification analyses to identify EEG correlates of the scope of visuo-spatial attention. Prior studies examining EEG correlates of local and global attention using standard analyses have not reported consistent ERP components that distinguished between locally- and globally-focused attention states (e.g., [33–38]). Although the variation in reported findings might be attributable to differences in specific tasks or stimulus properties, there are the additional possibilities that the critical neural correlates manifest as complex topographic patterns of EEG signals and/or considerable individual differences in those patterns mask any robust group-level effects. Either of these scenarios would reduce the sensitivity of typical ERP analyses, in which group-averaged data and a subset of electrodes are considered, whereas pattern-classification analysis would overcome these challenges as long as each individual’s neural correlate of attentional scope were reflected in a specific and consistent topography of EEG signals.

## Experiment 1: EEG correlates discriminating perceptual states

Using pattern-classification analyses, Experiment 1 allowed us to determine how EEG signals distinguished a variety of visual perceptual states. Based on the extensive previous EEG literature, we focus on three comparisons: left versus right stimulus location, face versus (non-face) Gabor stimuli and upright versus inverted faces (e.g., [20–23, 39]). All but the face versus non-face stimulus comparison are novel applications of pattern classification to EEG data, though unlike others, who presented cars as the non-face images, we presented Gabor stimuli.

To anticipate, in addition to replicating typical group-level ERP differences at established electrode sites, our pattern-classification analysis reliably differentiated left versus right stimulus locations, face versus Gabor stimuli, and upright versus inverted faces on a trial-by-trial basis. Specifically, the EEG pattern distinguishing stimuli presented in the left and right locations (irrespective of stimulus type) validated our particular implementation of pattern-classification analysis, by successfully identifying a simple scalp topography emphasizing posterior electrode sites with opposing weights for stimulus locations. Pattern classification also reliably decoded the perception of face versus Gabor stimuli and upright versus inverted faces on the basis of single-trial EEG.

### Methods

#### Participants

Eight individuals (5 women, age range = 21–34, *M* = 27 years) provided written informed consent to participate in the experiment (Northwestern University IRB approved the study; STU00013229). Seven individuals were naïve to the purposes of the experiment (paid $10/hr for their participation) and one was a trained observer (author AL; training produced no reliable difference or interactions on classification accuracy). All had normal or corrected-to-normal vision and were right-handed.

#### Apparatus

Stimulus presentation and manual response recording were controlled by Presentation software (www.neurobs.com; Version 12.199). A 20” Sony CRT monitor (60 Hz refresh rate and 1028 × 768 resolution) was used for visual stimulus presentation, at a viewing distance of 150 cm. Participants used a computer mouse to respond. EEG recording was carried out with a 68-channel (64 scalp and 4 facial electrodes, including a nose reference) active electrode Biosemi system (www.cortechsolutions.com), referenced to the nose, at a sampling rate of 1024 Hz.

#### Stimuli

**Fig 1:**
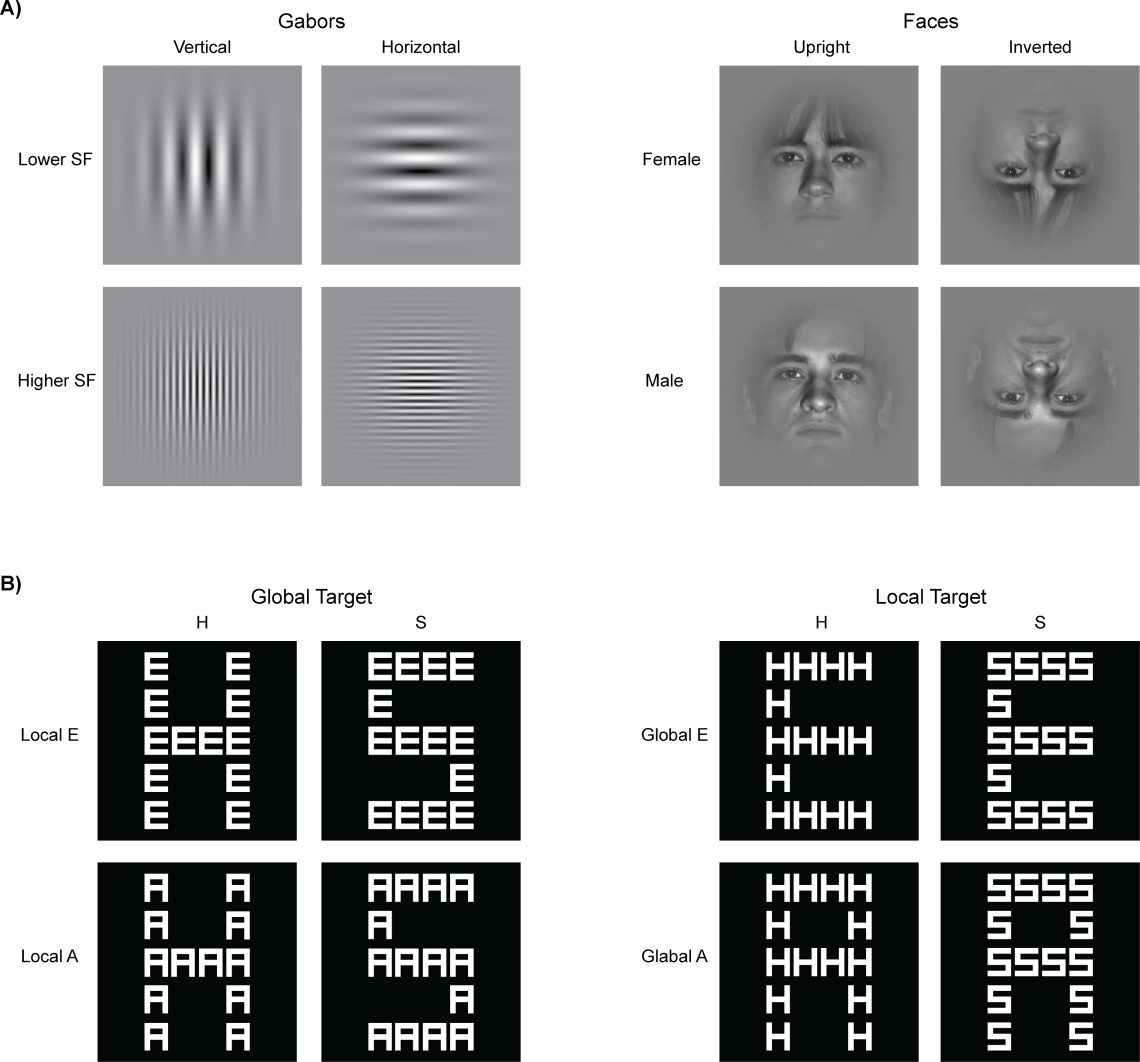
Stimuli. A) In Experiment 1, stimuli were presented individually either in the right or left visual field during passive viewing. SF = spatial frequency. B) In Experiment 2, stimuli were presented centrally and participants determined if the letter H or S was present, regardless of whether it appeared at the global or local level. Irrelevant distracter letters (E or A) were presented at the other level.

All stimuli were presented on a gray background (luminance = 11 cd/m^2^). Four different stimuli (4.5° by 4.5°) were presented individually, centered at 2.4° eccentricity to the left or right of a black (luminance = 0.5 cd/m^2^) central fixation dot (diameter = 0.2°). Two of the four stimuli were Gabor stimuli (0.97 Michelson contrast at peak contrast) with spatial frequencies of 7.9 cycles/degree (higher spatial frequency) and 1.3 cycles/degree (lower spatial frequency; Fig 1A, left). Note that these spatial frequencies are higher and lower (in log units) relative to the peak of the human contrast sensitivity function, and are approximately equivalently visible based on the published contrast-sensitivity functions (e.g., see the 1 Hz condition in [40]; see the relevant mesopic-photopic conditions in [41]; [42]).

The Gabor stimuli were oriented either vertically or horizontally. Thus, for the Gabor stimuli, the factorial stimulus design was Location (Left, Right) x Spatial frequency (High, Low) x Orientation (Vertical, Horizontal). The two remaining stimuli were faces (one female, one male) selected from the Extended Yale Face Database B (faces 17 and 32 from [43]). The face stimuli were presented upright or inverted (180° rotated in the picture plane). A Gaussian envelope was applied to the face stimuli to reduce image boundary edges (Fig 1A, right). Thus, for face stimuli, the factorial stimulus design was Location (Left, Right) x Identity/Gender (Female, Male) x Orientation (Upright, Inverted).

#### Design

EEG data were analyzed to determine the neural correlates of the following comparisons: left versus right location, face versus Gabor stimuli, and upright versus inverted faces. All conditions were collapsed over the other stimulus factors.

#### Behavioral procedure

Participants were instructed to fixate the central dot, and refrain from blinking or moving their eyes during passive viewing of the stimuli. The fixation dot appeared for 250 ms, followed by one of sixteen visual stimulus conditions (described above; Fig 1A) for 500 ms. Trials were separated by a 200–300 ms jittered inter-trial interval (duration was randomly selected from a uniform distribution in ~16 ms increments, due to monitor refresh rate), showing only the fixation. A 5-s break was presented every eight trials. Each block of 160 trials was composed of ten groups of 16 trials in which all 16 stimuli were presented in a randomized order. Six blocks of trials were run for a total of 960 trials. Participants took breaks between blocks as needed, and pressed the mouse button to initiate each block.

#### EEG signal processing

All channels were referenced to the nose. The raw EEG was bandpassed (0.1–30 Hz), and segmented into 1-s epochs (spanning 250 ms before to 750 ms after stimulus onset). A few channels (*M* = 2.25 channels per participant, *SD* = 2.25) were excluded from analysis due to poor scalp contact. Manual artifact rejection was conducted on the EEG signals from the remaining channels to remove epochs with blinks, eye movements and muscle activity. A mean total of 833 (*SD* = 111.4) stimulus epochs (or trials) per participant remained after artifact rejection, with a minimum of 69 trials/condition. Signal processing was carried out using Matlab (www.mathworks.com) and the EEGLAB toolbox [44].

#### Standard ERPs

**Fig 2:**
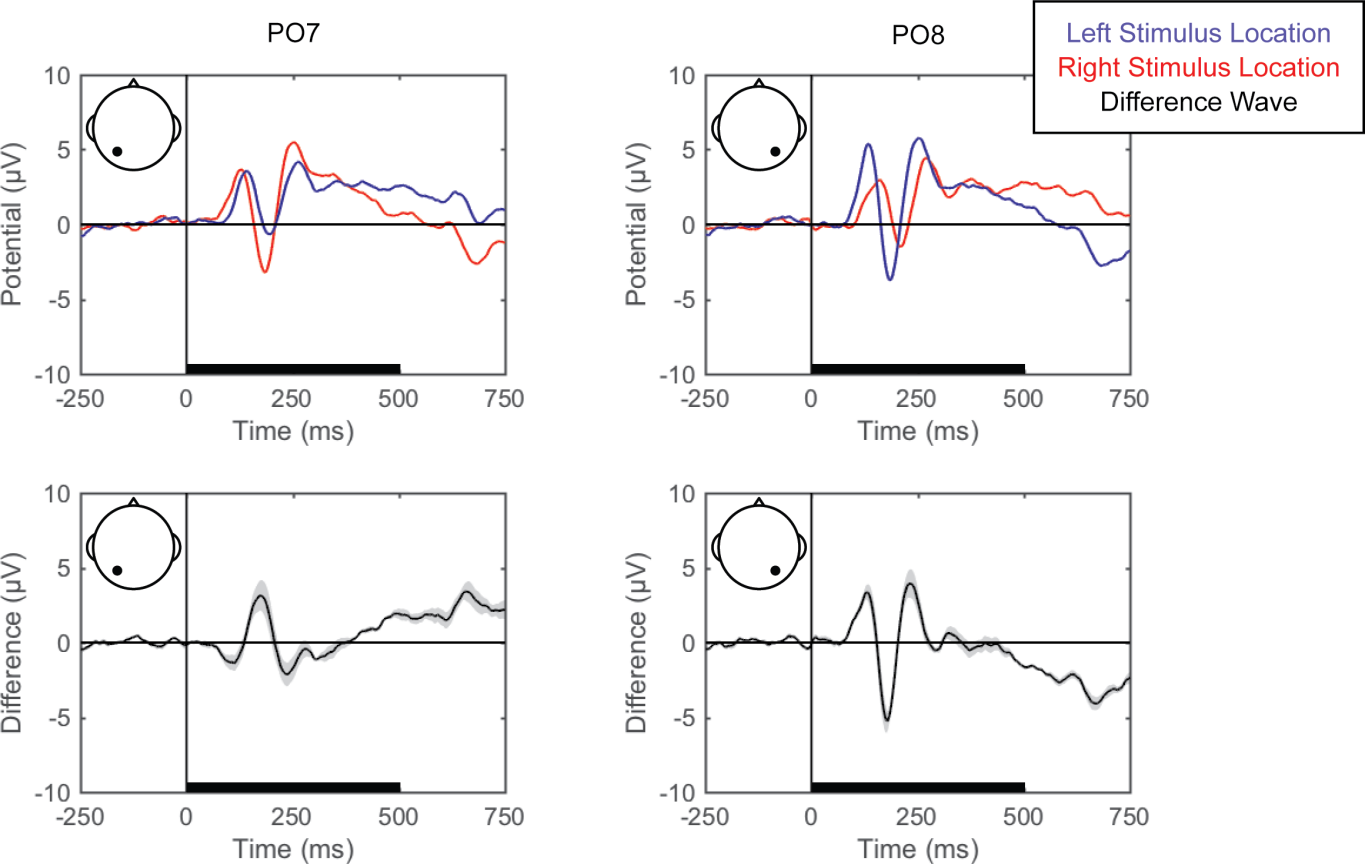
Grand average ERPs for right versus left stimulus location. Grand average ERPs are shown for electrodes PO7 (left) and PO8 (right), for the left (blue) and right (red) stimulus locations (top). The difference wave (black) with the within-subjects standard error (gray shading) are plotted (bottom). The black bars on the horizontal axes reflect stimulus duration.

**Fig 3:**
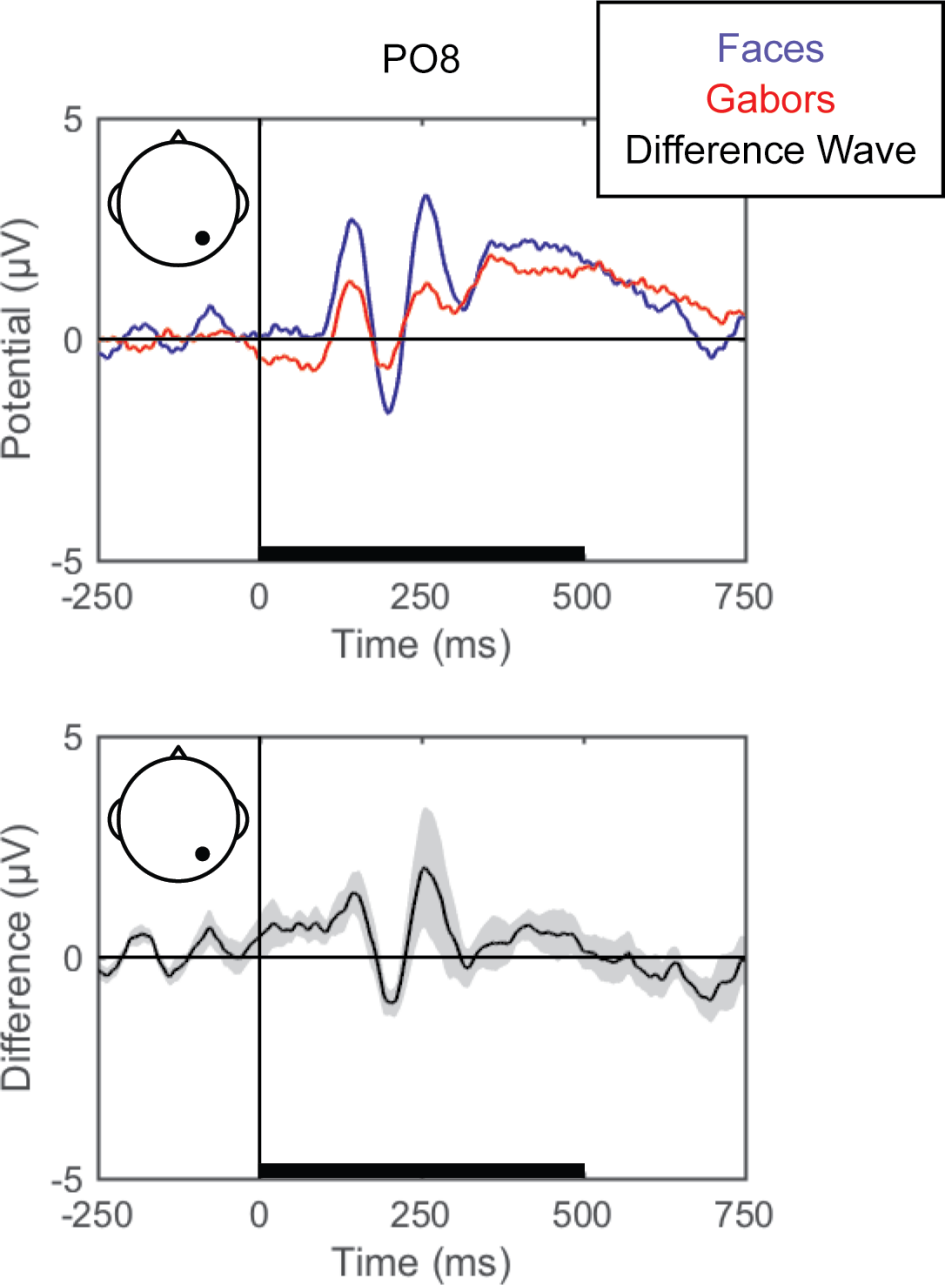
Grand average ERPs for faces versus Gabors. Grand average ERPs are shown for electrode PO8 for face (blue) and Gabor (red) stimuli (top). The difference wave (black) with the within-subjects standard error (gray shading) are plotted (bottom). The black bars on the horizontal axes reflect stimulus duration.

**Fig 4:**
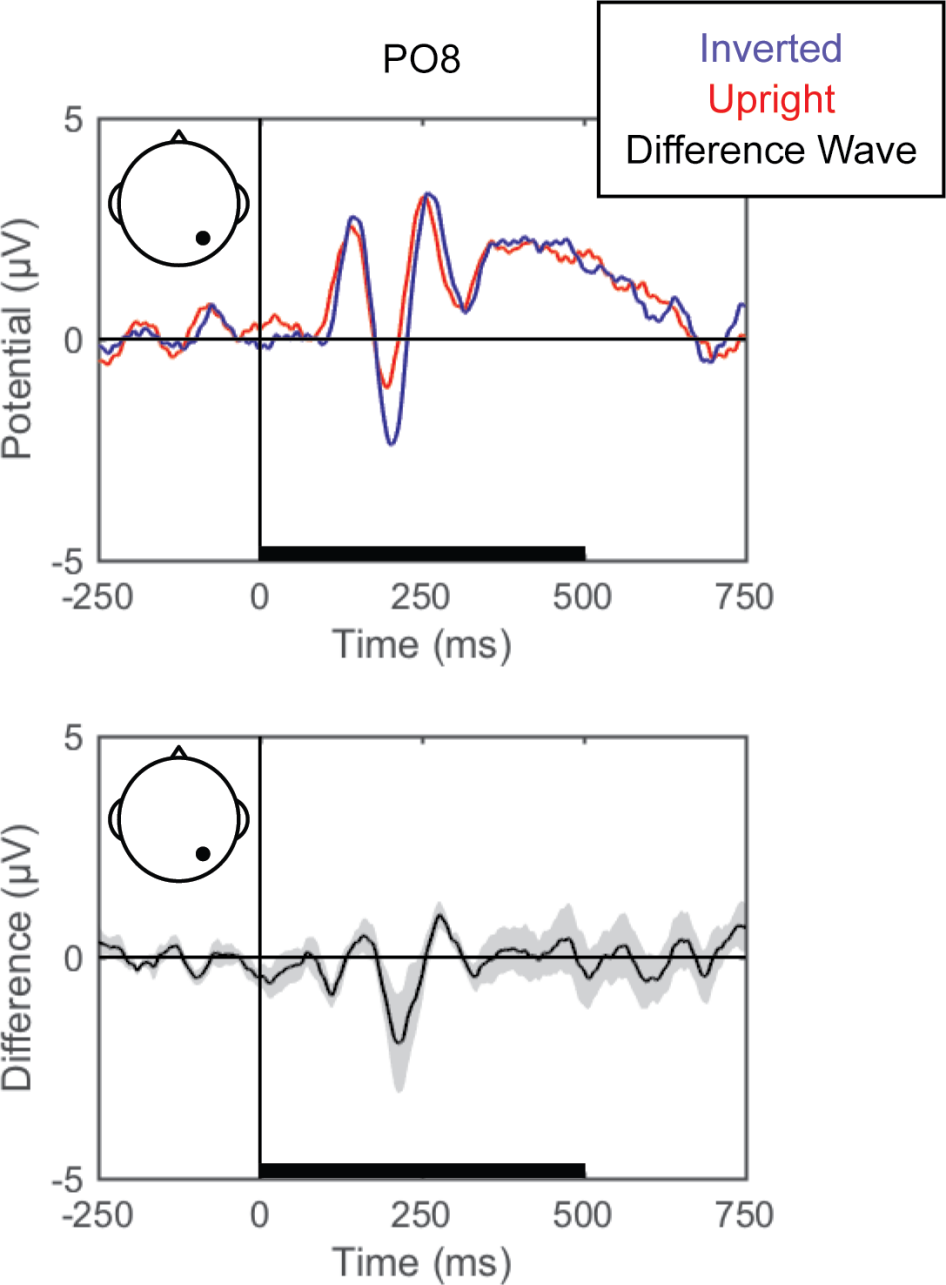
Grand average ERPs for inverted versus upright faces. Grand average ERPs are shown for electrode PO8 for inverted (blue) and upright (red) face stimuli (top). The difference wave (black) with the within-subjects standard error (gray shading) are plotted (bottom). The black bars on the horizontal axes reflect stimulus duration.

Grand averaged ERPs (EEG averaged across trials for each condition for each participant and then across participants) are shown for each of the perceptual comparisons in Fig 2, Fig 3 and Fig 4. For the left versus right stimulus location comparison, data from electrodes PO7 and PO8 are shown in Fig 2 to illustrate lateralization of processing. For the face versus Gabor comparison and the upright versus inverted face comparison, data from electrode PO8 are plotted (Fig 3 and Fig 4, respectively). Additionally, the grand averaged ERPs at all 64 scalp electrode sites are shown for each perceptual comparison in the supplemental materials (S1 Fig, S2 Fig and S3 Fig).

#### Pattern-classification analysis

For each participant, trial numbers were equated across conditions for each perceptual comparison via random subsampling from the condition with more trials. For example, if a participant had 410 face trials and 440 Gabor trials after artifact rejection, only 410 randomly-subsampled Gabor trials were submitted to classification analysis with all 410 face trials. A linear support vector classifier (http://www.csie.ntu.edu.tw/~cjlin/libsvm/) was then applied to single-trial EEG signals (μV) at each timepoint (~1 ms resolution) using, on average, 62 (*SD* = 2.25) electrodes as features. Continuing the example above, using all 820 trials, the first of 1024 timepoints (at -250 ms) μV value for each electrode would be submitted to a 10-fold cross-validation procedure. This cross-validation procedure iteratively divides the trials into 10 groups (in this example, 82 trials/group), trains the classifier to discriminate conditions based on 9 of the 10 trial groups (in this example, 738 trials), and tests the accuracy of the obtained EEG correlate for predicting conditions on the remaining trials (in this example, 82 trials). This cross-validation procedure yields a percent accurate classification for each of the 10 tests (% of individual trials accurately decoded), which are then averaged to produce the overall prediction accuracy. Thus, accuracy of 70% would represent 574/820 trials correctly classified. The whole process is repeated at each timepoint, separately for each participant, resulting in prediction accuracy for each participant, at each timepoint, from 250 ms pre-stimulus to 750 ms post-stimulus onset. Critically, the EEG data were not averaged over trials (single trials always served as instances), time or participant prior to classification, meaning that the prediction accuracy is derived at the single-trial, single-timepoint (~1 ms) level.

For each participant, we separately derived the electrode weights from the evenly-sampled dataset, revealing the relative importance of each electrode in discriminating between conditions. From these weights, we produced “importance maps,” or topographic maps of electrode weights at each timepoint for each participant. Each resultant importance map (for each participant at each timepoint) was normalized by dividing the individual electrodes’ weights by the standard deviation across channels.

To capture the general time course of informativeness of EEG correlates, we averaged the accuracy data across time and conducted group-level analyses. In doing so, we created a distribution for conducting inferential statistics and, although at the cost of temporal resolution, reduced type I error (for which 1024 timepoints is excessive). Specifically, for each perceptual comparison, we analyzed the average accuracy over successive 125-ms time bins (1000 ms divides evenly into eight 125-ms bins), which lies within the broad range of others’ analysis bins spanning tens to hundreds of ms (e.g., 10, 14, 15, 31). The 125-ms (i.e., 8 Hz) bin size is reasonable because it is commensurate with reported sampling rates of visual attention in the theta (4–8 Hz) and alpha (8–13 Hz) ranges (e.g., [45–52]). We evaluated the statistical reliability of pattern classification in the following way. We conducted a one-way repeated-measures ANOVA with temporal bin as the factor and participants as the random effect. If a significant main effect emerged, then we conducted Bonferroni-corrected *t*-tests against 50% (i.e., the α-level was adjusted to .00625) to identify the time bins in which pattern-classification analysis successfully identified an EEG correlate that distinguished the experimental conditions. In the figures, we present the accuracy averaged over individuals at the original ~1 ms resolution, overlaid with the time-averaged group accuracy mean and standard error. We also present the peak group-identified EEG correlate as a topography of averaged linear weights (i.e., the individual, ~1 ms resolution importance maps averaged over both the peak 125-ms period and individuals).

### Results

#### Left versus right stimulus location

**Fig 5:**
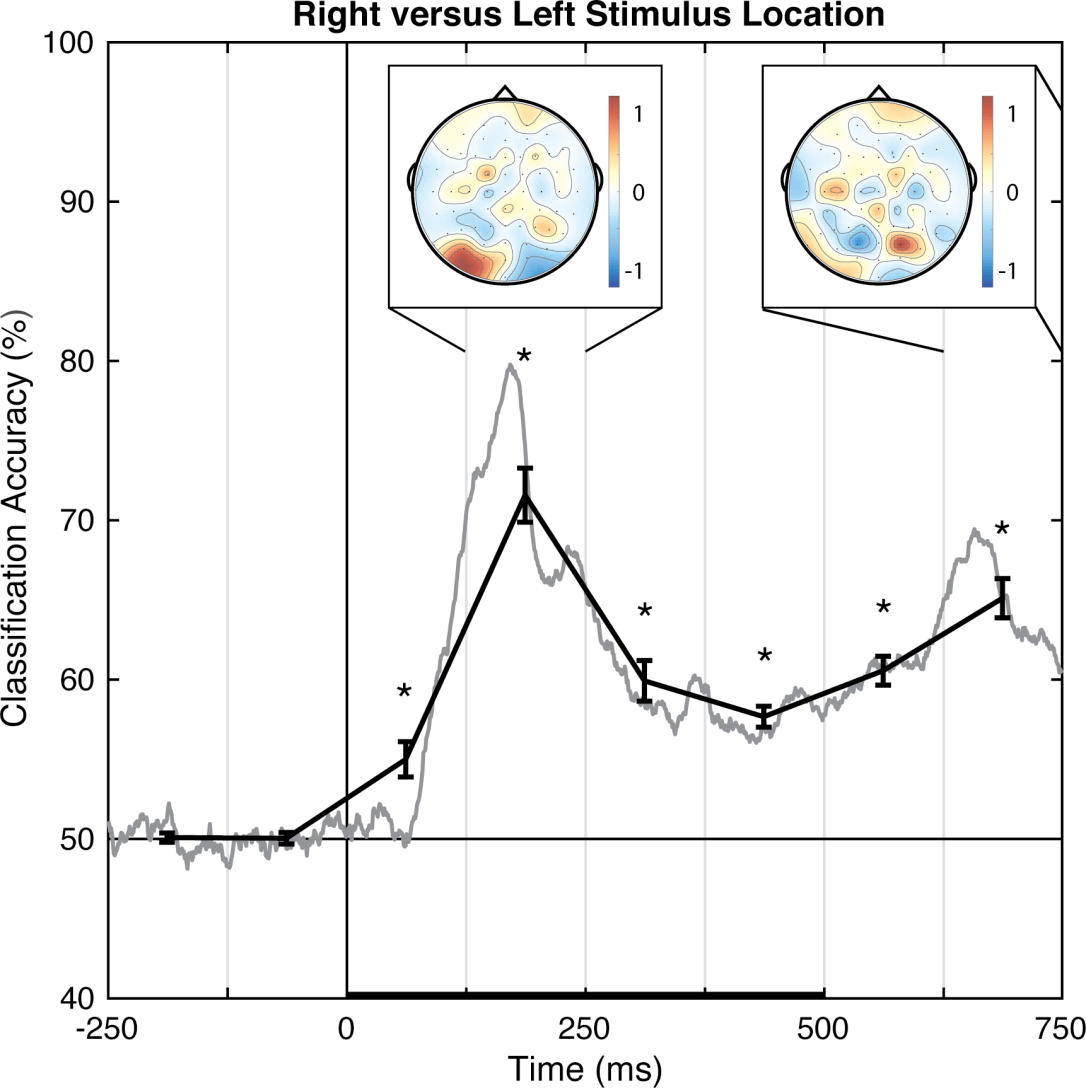
Group classification accuracy for right versus left stimulus location. The gray line shows the group-averaged accuracy at each time point. The black line shows the time-averaged accuracy for each 125-ms time bin (areas between vertical bars), on which inferential statistics were carried out (with within-subject standard errors). For the peak accuracy time bin, the heatmap shows the group-averaged electrode weights across the scalp, also averaged over 125-ms. Chance accuracy is 50% (black horizontal line), and the black horizontal bar on the lower axis reflects stimulus duration. * *p* < .00625 (Bonferroni-corrected α-level).

Pattern-classification analysis successfully distinguished left and right stimulus presentation locations, *F*(7,49) = 55.144, *p <* .001, $\eta_{p}^{2}$ = 0.887, with accuracy significantly above chance for all of the post-stimulus time bins, *t*s(7) > 4.50, *p*s < .00625, *d*s > 1.5 (Fig 5). Importantly, prediction accuracy was at chance for both pre-stimulus baseline bins, |*t|*s < 1, *p*s > .77, *d*s < 0.11. The prediction accuracy peaked over the 125–250 ms latency, with the associated topography of linear weights indicating that the EEG correlate of left versus right stimulus position discrimination emerges primarily from posterior electrode sites. Notably, the topography corresponding to the second peak of accuracy, occurring at the 625–750 ms latency, shows the opposite (left-right reversed) weight pattern. Because the stimulus disappeared 500 ms after stimulus onset, this may indicate location-specific neural adaptation, or the return of attention to the central fixation point (rightward return following a left stimulus and leftward return following a right stimulus). Additional research is necessary to understand the accompanying topographic change over time. However, at a minimum, the results indicate that EEG signals can distinguish between stimuli presented in left and right locations at ~70% accuracy on a trial-by-trial basis.

#### Faces versus Gabors

**Fig 6:**
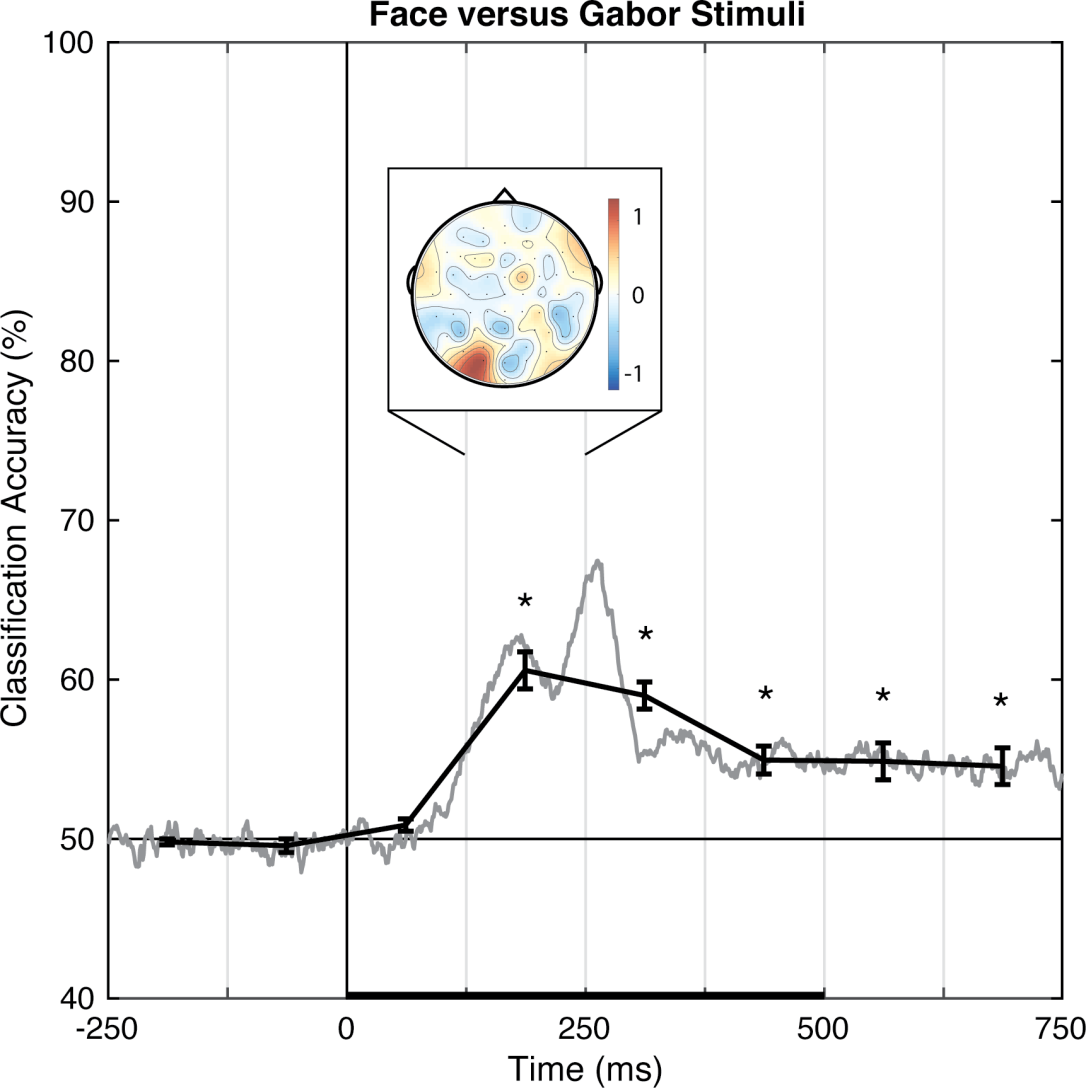
Group classification accuracy for face versus Gabor stimuli. The gray line shows the group-averaged accuracy at each time point. The black line shows the time-averaged accuracy for each 125-ms time bin, on which inferential statistics were carried out (with within-subject standard errors). For the peak accuracy time bin, the heatmap shows the group-averaged electrode weights across the scalp, also averaged over 125 ms. Chance accuracy is 50% (black horizontal line), and the black horizontal bar on the lower axis reflects stimulus duration. * *p* < .00625 (Bonferroni-corrected α-level).

Pattern-classification analysis robustly distinguished face and Gabor stimuli, *F*(7,49) = 26.963, *p <* .001, $\eta_{p}^{2}$ = 0.794, with accuracy significantly above chance for all time bins 125 ms and later, *t*s(7) > 3.9, *p*s < .00625, *d*s > 1.4 (Fig 6). Again, prediction accuracy was at chance for both pre-stimulus baseline bins, |*t|*s < 1, *p*s > .35, *d*s < 0.4, and failed to meet significance for the 0–125 ms time bin, *t*(7) = 2.255, *p* = .059, *d* = 0.797. The accuracy peaked over the 125–250 ms latency, consistent with the timeframe in which the N170 face-sensitive ERP component is typically reported. The associated topography of linear weights is complex, but the left posterior sites emerged as especially informative (or, at least, consistently informative across participants).

#### Upright versus inverted faces

**Fig 7:**
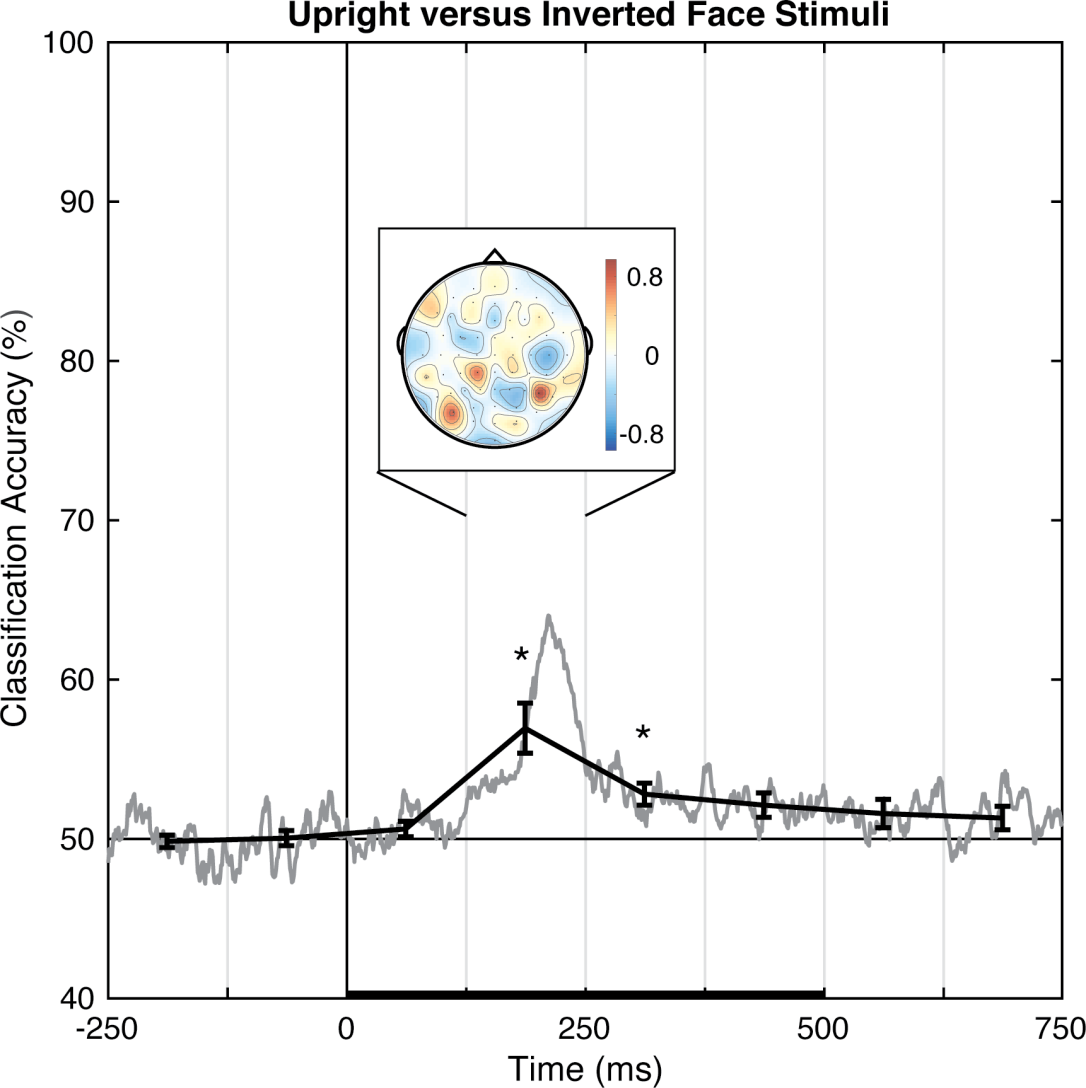
Group classification accuracy for upright versus inverted face stimuli. The gray line shows the group-averaged accuracy at each time point. The black line shows the time-averaged accuracy for each 125-ms time bin, on which inferential statistics were carried out (with within-subject standard errors). For the peak accuracy time bin, the heatmap shows the group-averaged electrode weights across the scalp, also averaged over 125 ms. Chance accuracy is 50% (black horizontal line), and the black horizontal bar on the lower axis reflects stimulus duration. * *p* < .00625 (Bonferroni-corrected α-level).

Pattern-classification analysis successfully distinguished upright and inverted faces, *F*(7,49) = 9.072, *p <* .001, $\eta_{p}^{2}$ = 0.564, with accuracy significantly above chance for the 125 to 375 ms time bins, *t*s(7) > 4.0, *p*s *<* .00625, *d*s > 1.4, and failing to meet correction levels for the following time bin, *t*(7) = 2.761, *p* < .05, *d* = 0.976 (Fig 7). Again, prediction accuracy was at chance for both pre-stimulus baseline bins, |*t|*s < 1, *p*s > .71, *d*s < 0.2, and was unreliable for the last two time bins, *t*s(7) ≈ 1.8, *p*s ≈ .12, *d*s ≈ 0.63. The accuracy peaked over the 125–250 ms latency, which is coarsely consistent with the timeframe in which the N170 face inversion-sensitive ERP component is typically reported. The associated topography of linear weights is complex, but posterior sites emerged as especially informative across participants.

### Discussion

Pattern-classification analyses identified linear topographies of EEG signals that successfully distinguish, on a trial-by-trial basis, visual stimuli presented in left versus right locations, face versus Gabor stimuli, and upright versus inverted faces. Notably, the classification of face perception was consistent with the established timing and posterior topography of the N170 ERP findings. Furthermore, the results replicate and extend other researchers’ successes in decoding the perception of face versus non-face stimuli based on trial-by-trial analyses of EEG (e.g., [5–7, 12–13]). Having established that our particular pattern-classification procedure is a viable approach to decoding EEG patterns for different perceptual states, we turned to the novel question of whether the analyses could decode the local or global scope of visual attention.

## Experiment 2: EEG correlates discriminating local versus global attentional states

In Experiment 2, we examined EEG correlates for the scope of visual spatial attention. In particular, we used pattern-classification analyses to determine whether a linear topography of EEG signals was able to distinguish locally- from globally-focused attentional states on a trial-by-trial basis. To do so, participants were assigned two target letters (H and S), and were asked to identify which of the two letters was present in a hierarchical stimulus, and to respond with the assigned finger. Only one target was present in any single stimulus, and the target was equally likely to be presented at the local or global level of the hierarchical stimulus (Fig 1B). Using this design, participants must attend either locally, to accurately identify a small repeated target letter, or globally, to accurately identify a large single target letter.

### Methods

Only methods differing from those described in Experiment 1 are detailed below.

#### Participants

Fifteen individuals (7 women, age range 18–44, *M* = 27 years) provided written informed consent to participate. All were naïve to the purposes of the experiment, except three unpaid trained observers (authors AL and AS; and a colleague; training produced no reliable difference or interactions on classification accuracy). All participants had normal or corrected-to-normal vision and 12 were right-handed. Two of the participants (AL and one other) also participated in Experiment 1 (see S4 Fig, S5 Fig, S6 Fig, S9 Fig and S10 Fig).

#### Apparatus

Participants used a number pad for responses.

#### Stimuli

Participants viewed white (37.5 cd/m^2^) hierarchical stimuli presented against a black background (0.5 cd/m^2^) at a viewing distance of 135 cm. Within each hierarchical stimulus the global letter subtended 1.1° x 1.7°, and each local letter subtended 0.2° x 0.3°. There were eight hierarchical stimuli from the factorial combination of the target letter, H or S, appearing at the local or global level, and the irrelevant letter, A or E, appearing at the other level (Fig 1B). A central fixation dot was presented in either red or white (see Behavioral procedure below).

#### Design

EEG data were analyzed for left- versus right-finger responses, and local versus global attention.

#### Behavioral procedure

Participants were instructed to identify which of two target letters (H or S) were presented, regardless of the level (global or local). Participants were assigned one button for each target letter, with response hand counter-balanced across participants (seven participants responded “S” with their left-hand and “H” with their right-hand, and the other eight did the reverse). A red fixation dot appeared for 2048 ms, followed by one of eight hierarchical stimuli for 100 ms. Trials were separated by a 1152–1252 ms randomly jittered inter-trial interval (durations were randomly selected from a uniform distribution in ~16 ms increments, due to monitor refresh rate), showing only the white fixation dot. Participants were instructed to fixate the dot at the center of the monitor, and refrain from blinking or moving their eyes during presentation of the red fixation and hierarchical stimulus. A 5-s break was given every four trials. Each block of 96 trials was composed of 12 groups of 8 trials in which all 8 stimuli were presented in a randomized order. Five blocks of trials were run for a total of 480 trials. Participants took breaks between blocks as needed, and made a button press to initiate each block.

#### EEG signal processing

On average, three (SD = 2.4) noisy channels were excluded from EEG analyses. After excluding artifacts and inaccurate trials, an average of 404 (SD = 62) total trials remained for each participant, with a minimum of 136 trials per condition. Again, EEG traces were time-locked to the stimulus onset.

#### Standard ERPs

**Fig 8:**
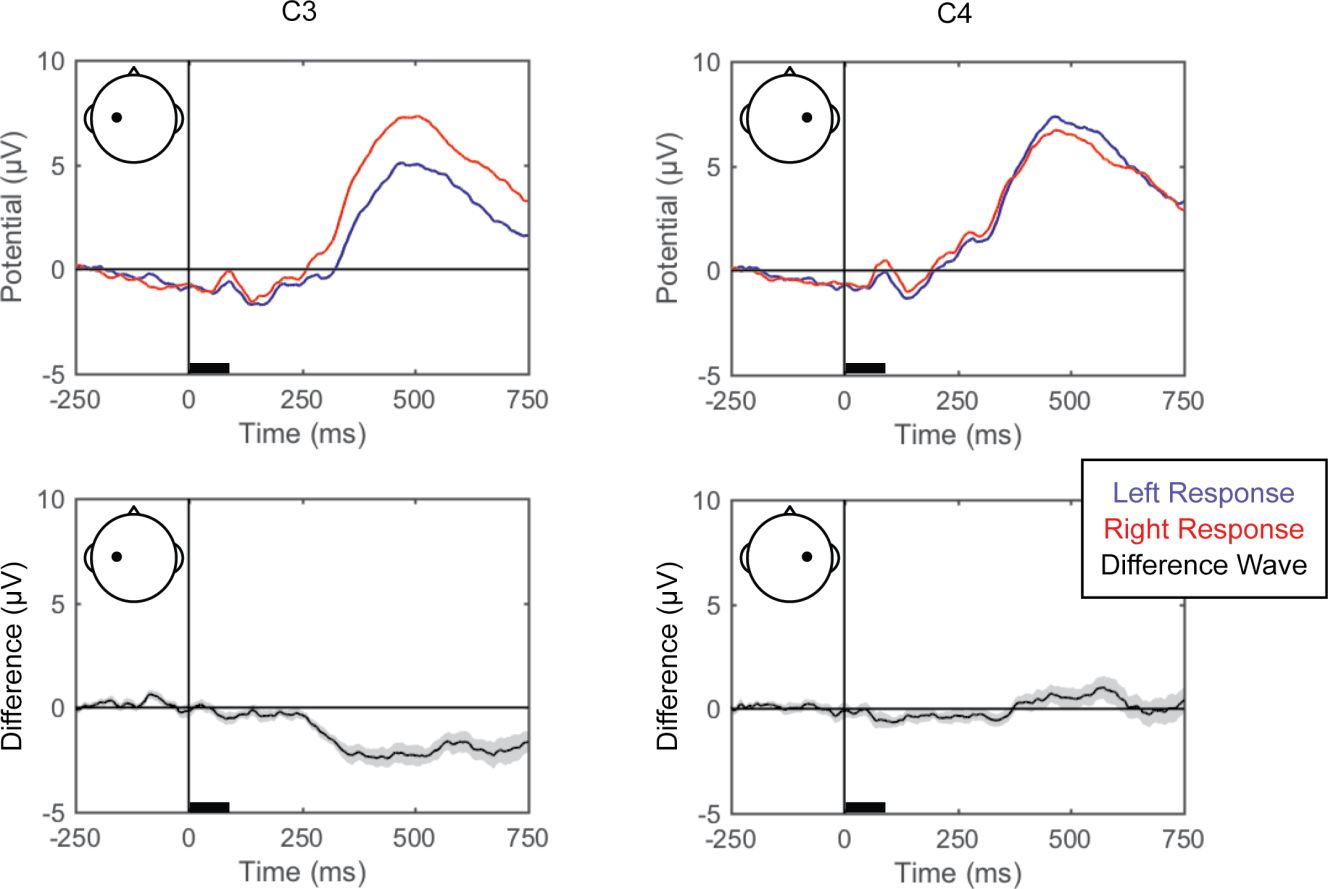
Grand average ERPs for right versus left responses. Grand average ERPs are shown for electrodes C3 (left) and C4 (right), for the left (blue) and right (red) responses (top). The difference wave (black) with the within-subjects standard error (gray shading) are plotted (bottom). The black bars on the horizontal axes reflect stimulus duration.

**Fig 9:**
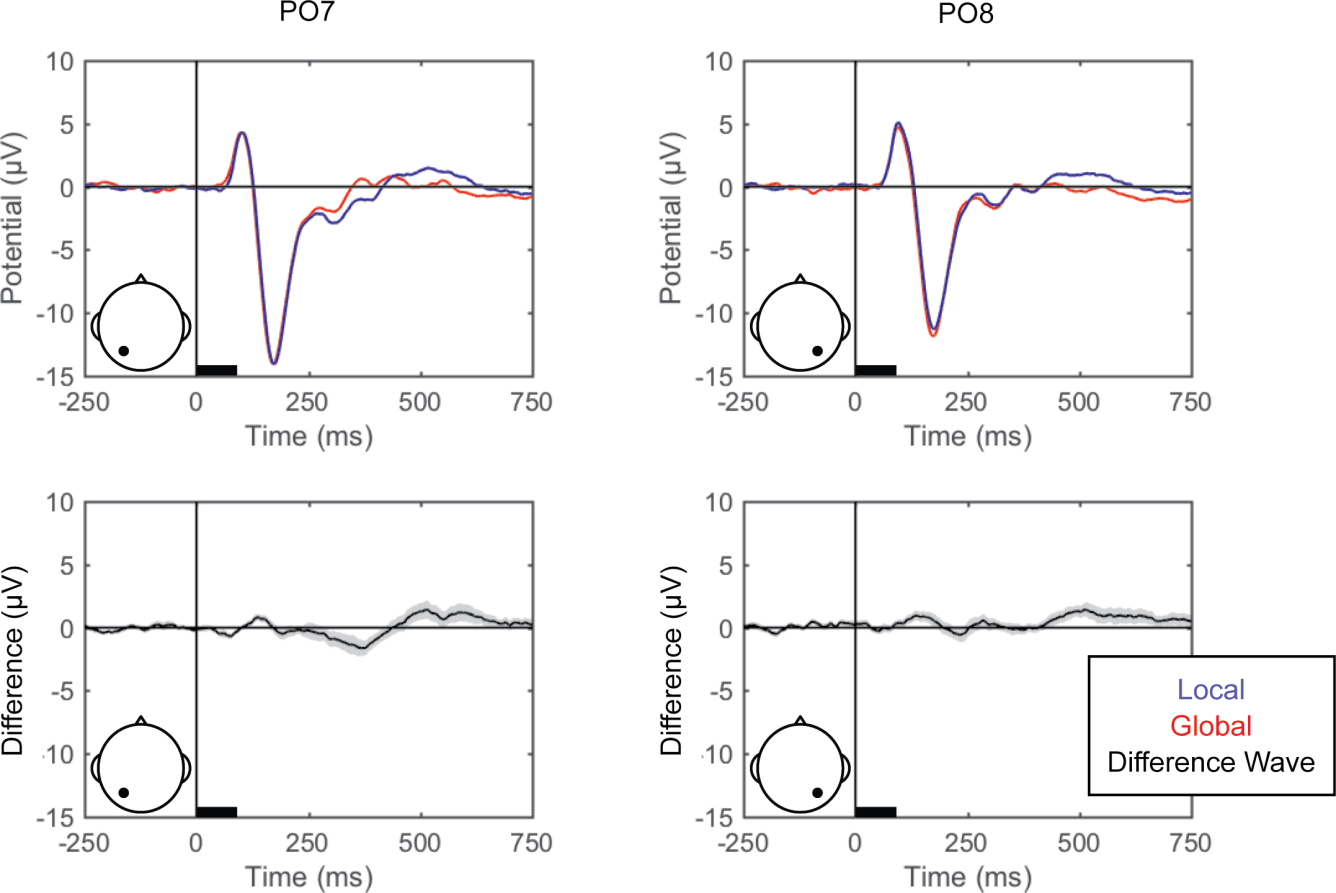
Grand average ERPs for local versus global attention. Grand average ERPs are shown for electrodes PO7 (left) and PO8 (right), for local (blue) and global (red) attention (top). The difference wave (black) with the within-subjects standard error (gray shading) are plotted (bottom). The black bars on the horizontal axes reflect stimulus duration.

Grand averaged ERPs (averaged across trials and then across participants) for the left and right finger responses (from C3 and C4 to illustrate lateralization of processing), and local and global attention (from PO7 and PO8) are shown in Fig 8 and Fig 9, respectively, and for all scalp electrode sites in the supplemental materials (S4 Fig and S5 Fig).

### Results

**Fig 10:**
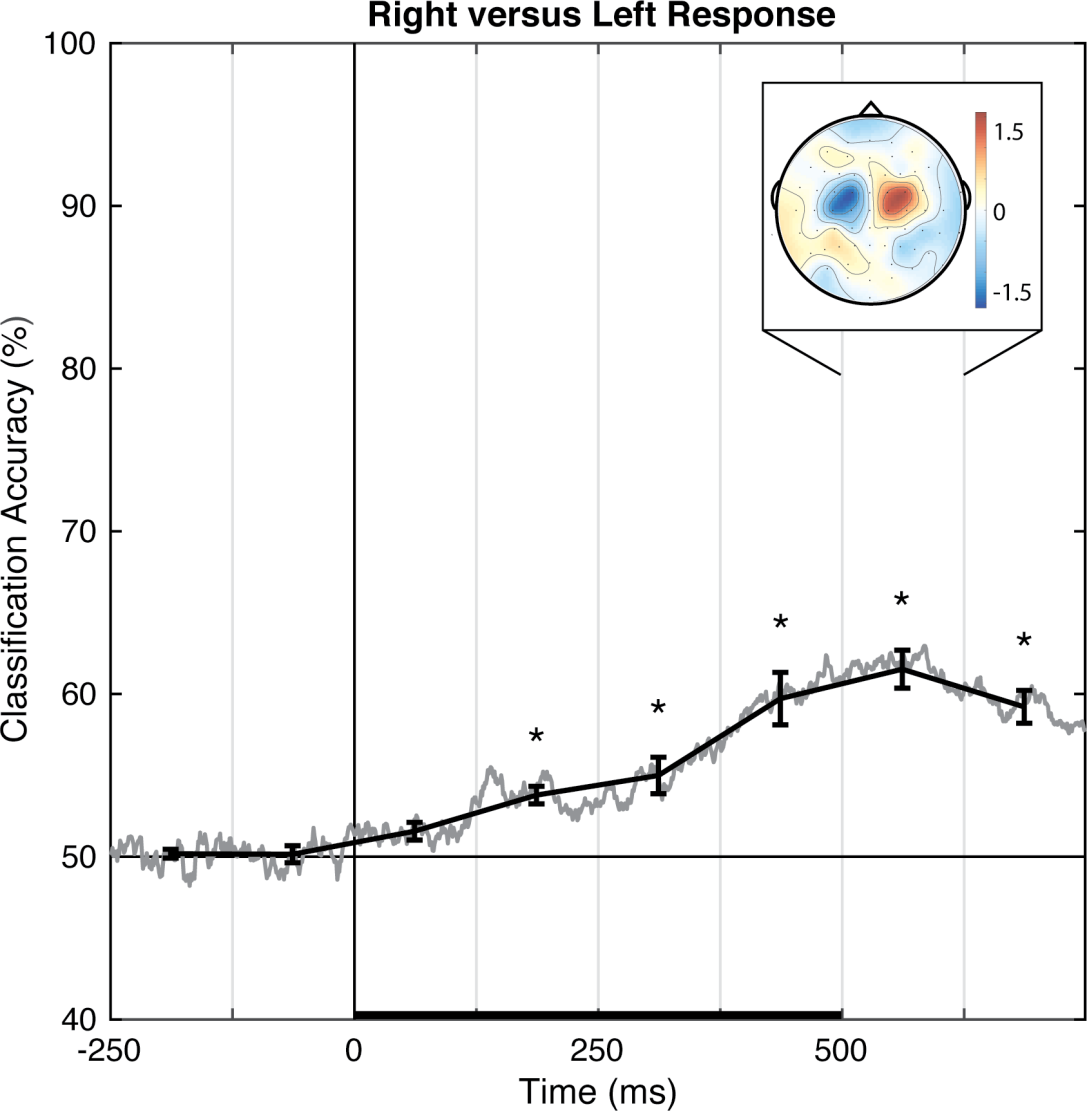
Group classification accuracy for left versus right response. The gray line shows the group-averaged accuracy at each time point. The black line shows the time-averaged accuracy for each 125-ms time bin, on which inferential statistics were carried out (with within-subject standard errors). For the peak accuracy time bin, the heatmap shows the group-averaged electrode weights across the scalp, also averaged over 125 ms. Chance accuracy is 50% (black horizontal line), and the black horizontal bar on the lower axis reflects stimulus duration. * *p* < .00625 (Bonferroni-corrected α-level).

As a methodological validation, we examined the current dataset for a linear topography of EEG signals distinguishing left from right button presses, a manipulation that has a well-established and robust contralateral central superior ERP signature (Fig 8; the lateral readiness potential, LRP; e.g., [53]). Pattern-classification analysis successfully distinguished left- and right-finger responses, *F*(7,98) = 28.619, *p <* .001, $\eta_{p}^{2}$ = 0.672, with the accuracy significantly above chance for all time bins after 125 ms, *t*s(14) > 4.45, *p*s *≤* .001, *d*s > 1.1, and failing to meet correction levels for the 0–125 ms post-stimulus time bin, *t*(14) = 2.845, *p <* .05, *d* = 0.734 (Fig 10). Prediction accuracy was at chance for both pre-stimulus baseline bins, |*t|*s < 1, *p*s > .51, *d*s < 0.2. The left-right response classification accuracy peaked over 500–625 ms, and the associated topography of linear weights clearly indicates that central electrode sites just lateral to midline primarily contribute to the EEG correlate of left- versus right-finger responses.

**Fig 11:**
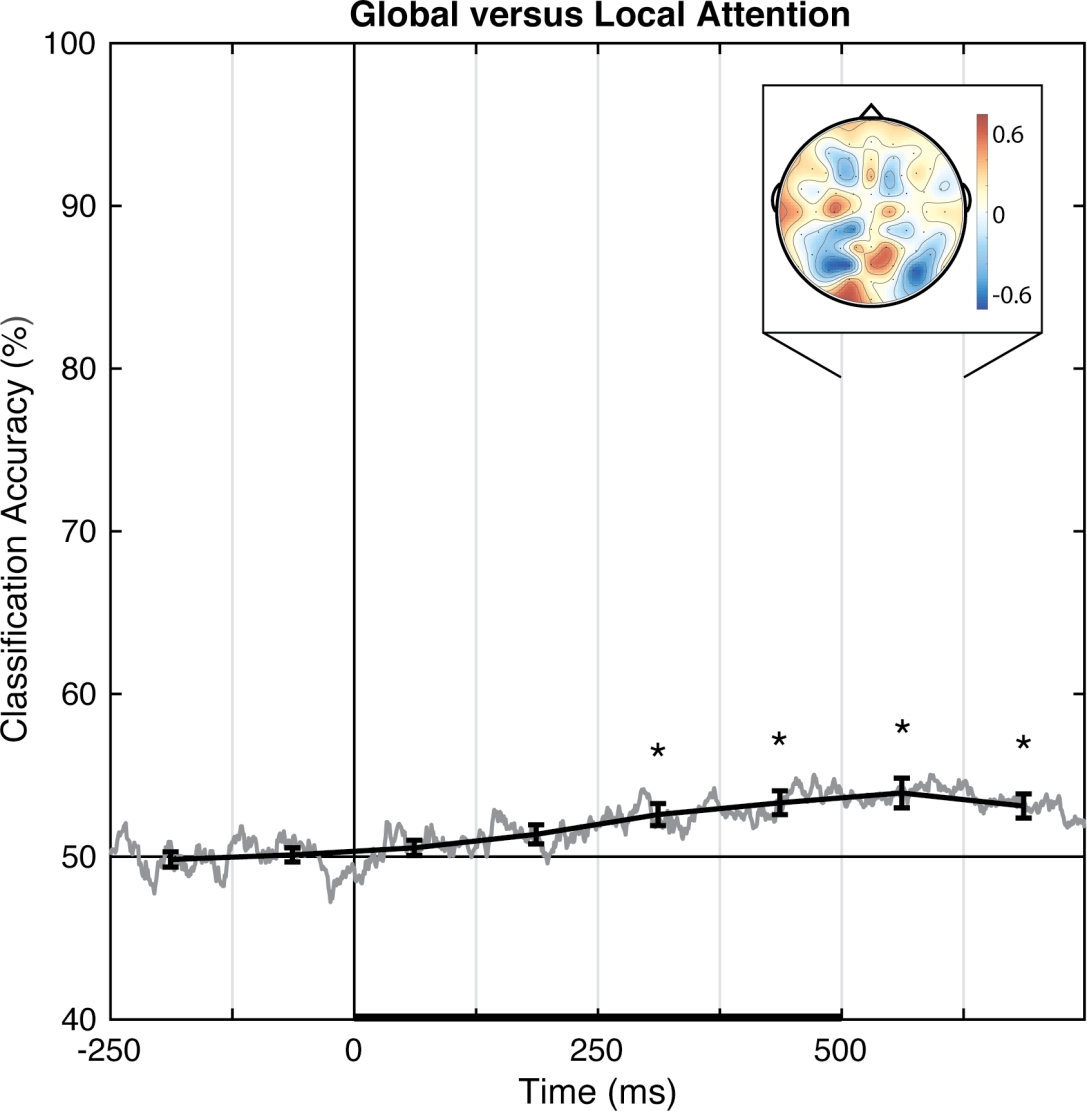
Group classification accuracy for global versus local attention. The gray line shows the group-averaged accuracy at each time point. The black line shows the time-averaged accuracy for each 125-ms time bin, on which inferential statistics were carried out (with within-subject standard errors). For the peak accuracy time bin, the heatmap shows the group-averaged electrode weights across the scalp, also averaged over 125 ms. Chance accuracy is 50% (black horizontal line), and the black horizontal bar on the lower axis reflects stimulus duration. * *p* < .00625 (Bonferroni-corrected α-level).

**Fig 12:**
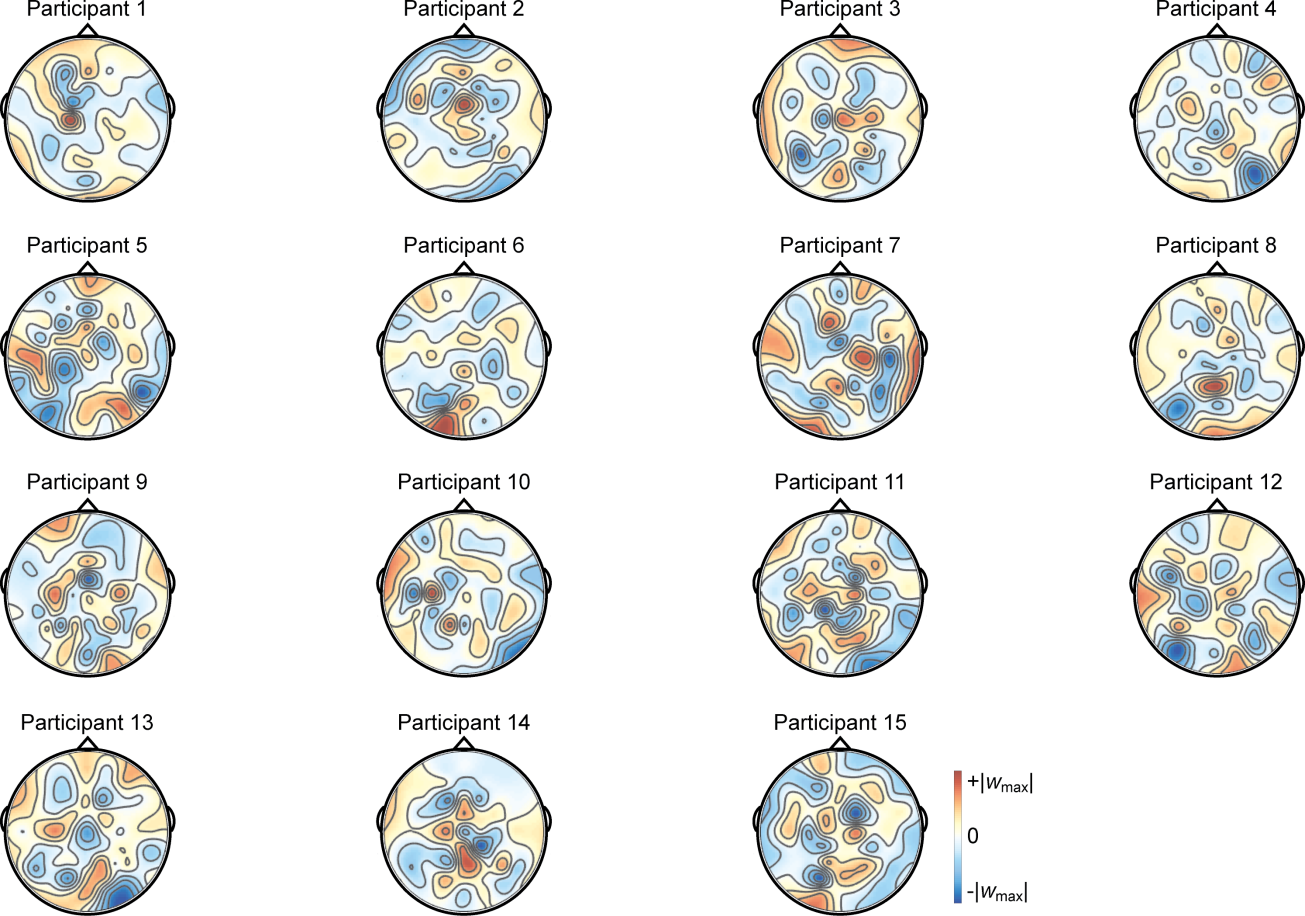
Individual participants’ importance maps for global versus local attention. The importance maps show each participant’s average weights over the 500–625 ms time bin (the 125-ms time bin showing peak group classification). For each individual, the color scale maximum and minimum are set to the positive and negative absolute maximum weight value, to be symmetric about 0. The most informative electrodes are reflected in intense blue or red, with white as least informative.

Pattern-classification analysis also successfully distinguished locally- from globally-focused attentional states, *F*(7,98) = 9.619, *p <* .001, $\eta_{p}^{2}$ = 0.407, with the accuracy significantly above chance for all bins after 250 ms, *t*s(14) > 3.8, *p*s ≤ .002, *d*s > 0.98 (Fig 11). Prediction accuracy was at chance for both pre-stimulus baseline bins, |*t|*s < 1, *p*s > .70, *d*s < 0.10, from 0–125 ms post-stimulus, *t*(14) = 1.14, *p* = .27, *d* = 0.294, and failed to meet the corrected threshold for 125–250 ms, *t*(14) = 2.339, *p* < .05, *d* = 0.604. Accuracy peaked over 500–625 ms. The associated group topography of linear weights is complex, perhaps indicative of variability between individuals (Fig 12), although at the group level posterior regions emerge as relatively more informative than anterior regions.

### Discussion

In Experiment 2, pattern-classification analyses identified linear topographies of EEG signals that successfully distinguish, on a trial-by-trial basis, left versus right responses as well as locally-focused versus globally-focused attention. The decoded topography for left versus right responses was consistent with well-established ERP results showing a lateralized and opposing central superior signature. Notably, pattern classification reliably distinguished between locally-focused versus globally-focused attention, though the scalp topography suggests a complex relationship across electrodes at the group level, which may be the result of individual variability in decoded linear topographies of EEG (Fig 12). Critically, to our knowledge, this is the first report to show the decoding of the *scope* of attention using pattern-classification analyses of EEG signal.

## General discussion

Pattern-classification analyses have recently been applied to multi-channel EEG signals to increase the sensitivity for identifying EEG correlates of perceptual, attentional, and behavioral states (e.g., [5–7, 10, 12–13, 54]). Instead of having to rely on data inspection and/or prior research to select a specific cluster of electrodes for which ERPs are compared between experimental conditions, linear pattern-classification analysis algorithmically identifies a multivariate topography of EEG signals that most effectively distinguishes experimental conditions on a trial-by-trial basis. Thus, pattern-classification analysis lends itself to differentiating cognitive processes which are represented by more complex spatiotemporal patterns of activity, compared with more modular systems and analyses which depend entirely on group effects. Here, we extend prior EEG applications of pattern-classification analyses toward decoding perceptual states, namely spatial and face perception, to confirm the correspondence between our particular application of pattern classification and well-established ERPs. Secondly, we examined whether these pattern-classification methods could identify EEG correlates of the scope of visual attention (i.e., locally- versus globally-focused attention), that do not a have a well-defined differentiating ERP correlate.

In Experiment 1, the perceptual state classifiers decoded visual stimulus hemifield, faces versus Gabors, and upright versus inverted faces. The importance maps from these classifiers were generally consistent with well-established ERP correlates of spatial perception and face perception. Presentation of stimuli in the left versus right visual location produced the strongest weights over posterior electrodes (Fig 5), and the classifier was most successful over time windows that similarly showed divergence in the ERPs (Fig 2, Fig 3 and Fig 4). The EEG correlates that distinguish between the perceptual states of seeing faces versus Gabors or upright versus inverted faces show complex linear topographies (Fig 4 and Fig 5), but posterior scalp sites emerge as the most informative in both cases, as is commonplace for visual ERPs (S2 Fig and S3 Fig). Furthermore, face perception classification results peak over a similar time course as the differences in the N170 ERP component (Fig 2, Fig 4 and Fig 5). Thus, these results support the effectiveness of our technique for identifying EEG correlates that predict perceptual states varying on a trial-by-trial basis. It is interesting to note that for all successful perceptual pattern classifiers (left versus right locations, faces versus non-face Gabors, or upright versus inverted faces), classification accuracy—the amount of relevant information present in the linear topography of EEG signals—peaked in the post-stimulus interval of 125–250 ms. This consistent latency may suggest that, for distinguishing perceptual states, linear topography of EEG signals might be suitable for revealing neural correlates that include the initial volley of feedback signals from higher-level visual areas (e.g., [55–57]).

Our pattern-classification analysis also succeeded in identifying a distributed EEG signature of the scope of attention. EEG patterns distinguished locally- and globally-focused states of attention beginning 125–250 ms after stimulus onset, when perceptual classification peaked in Experiment 1, and were maximally discriminable 500–625 ms post-onset. Thus, using EEG data, the scope of attention initially becomes predictable over a similar timecourse as perception, but is most distinguishable hundreds of ms after the stimulus onset. The average response time was 604 ms, which suggests that the scope of attention appropriate for each trial continues to develop through the time of making an overt manual response. The identified topography of linear weights is complex, apparently dominated by four posterior scalp sites, including a pair of contiguous sites (red, Fig 11) near the midline and a pair of lateral sites (blue, Fig 11) that make opposing contributions during the 500–625 ms time window. No single contiguous cluster of electrode sites distinguishes local from global attention across the group, possibly explaining why prior ERP markers have not converged onto a consistent ERP component that discriminates between the two attention states. Importantly, unlike ERP results that describe group- and trial-averaged neural responses, the present approach does allow us to predict the scope of attention for individual subjects on a trial-by-trial basis.

Classifiers trained to predict an individual’s perceptual and/or attentional state on a single-trial basis, either without an overt behavioral response (as in Experiment 1) or well before the response (as in Experiment 2), could be adopted for interventions via HCI (human-computer interfacing). Here, we established a simple classification analysis routine with minimal EEG data reduction and processing, and to explore, in Experiment 2, an EEG signal that has stubbornly eluded ERP and group-level analysis—the electrophysiological markers differentiating local and global attention. The latter point, that single-subject, single-trial pattern-classification analysis successfully differentiated the scope of attention, is novel and important because it establishes that classifiers may differentiate EEG signals successfully even where ERP analyses have failed. In future exploratory studies, it may be possible to even further increase prediction accuracy with additional data processing (by, e.g., refining temporal windows, averaging signal over those time windows, applying Bayesian statistics as Treder [46] did).

In summary, we have shown that topographic patterns of EEG signals predict perceptual and attentional states on a trial-by-trial basis. The obtained topographies of linear weights were relatively straightforward for distinguishing left and right visual presentations (marked by the lateralized and opposing contributions of posterior electrode sites), for distinguishing left and right finger responses (marked by the lateralized and opposing contributions of central electrode sites), and for distinguishing faces and Gabors (marked by the left posterior electrode sites), but were more complex for distinguishing locally- and globally-focused attention states and upright versus inverted faces. It is possible that further data processing and/or appropriate non-linear weighting of electrode sites would reveal EEG correlates that predict perceptual and attentional states with even greater accuracy (see, e.g., [46, 58]). It is also possible that non-linear transformations of electrode sites using relatively assumption free methods such as current-source-density transformation (e.g., [54, 59–60]) or second-order blind-source-separation transformation (e.g., [61]), which more accurately reflect the underlying neural generators of EEG signals, may enable pattern-classification analyses to identify EEG correlates of perceptual and attentional states with even greater sensitivity. Multivariate approaches applied to EEG have the exciting potential to reveal the spatial and temporal properties of neural systems that underlie complex cognitive states that may otherwise be obscured in traditional univariate approaches.

## Supporting information

S1 Fig Grand average ERPs for stimuli presented in the right versus left visual stimulus location in Experiment 1. (TIFF)

S2 Fig Grand average ERPs for face versus Gabor stimuli in Experiment 1. (TIFF)

S3 Fig Grand average ERPs for upright versus inverted face stimuli in Experiment 1. (TIFF)

S4 Fig Individual classification accuracy for right versus left visual stimulus location in Experiment 1. Note that participant 1 was a trained observer. Participant 1 and 4 also participated in Experiment 2.
(TIF)

S5 Fig Individual classification accuracy for face versus Gabor stimuli in Experiment 1. Note that participant 1 was a trained observer. Participant 1 and 4 also participated in Experiment 2.
(TIF)

S6 Fig Individual classification accuracy for upright versus inverted face stimuli in Experiment 1. Note that participant 1 was a trained observer. Participant 1 and 4 so participated in Experiment 2.
(TIF)

S7 Fig Grand average ERPs for right versus left response in Experiment 2. (TIFF)

S8 Fig Grand average ERPs for global versus local attention in Experiment 2. (TIFF)

S9 Fig Individual classification accuracy for right versus left response in Experiment 2. Note that participants 1, 2 and 14 were trained observers. Participant 1 and 11 also participated in Experiment 1 (Participants 1 and 4, respectively, in Experiment 1).
(ZIP)

S10 Fig Individual classification accuracy for global versus local attention in Experiment 2. Note that participants 1, 2 and 14 were trained observers. Participant 1 and 11 also participated in Experiment 1 (Participants 1 and 4, respectively, in Experiment 1).
(ZIP)
