# Supplementary figures and images for: Pattern classification of EEG signals reveals perceptual and attentional states
Source: PLoS One. 2017 Apr 26;12(4):e0176349. doi: 10.1371/journal.pone.0176349 (PMC5405963; doi:10.1371/journal.pone.0176349)

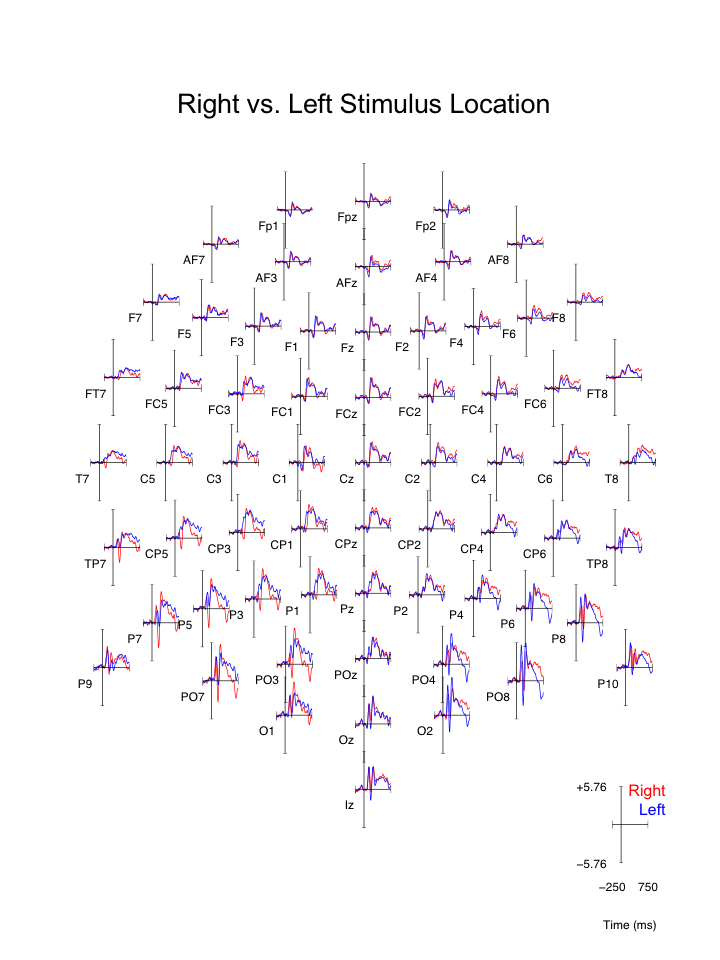

Supplement: S1 Fig — (TIFF) [file pone.0176349.s001.tiff]

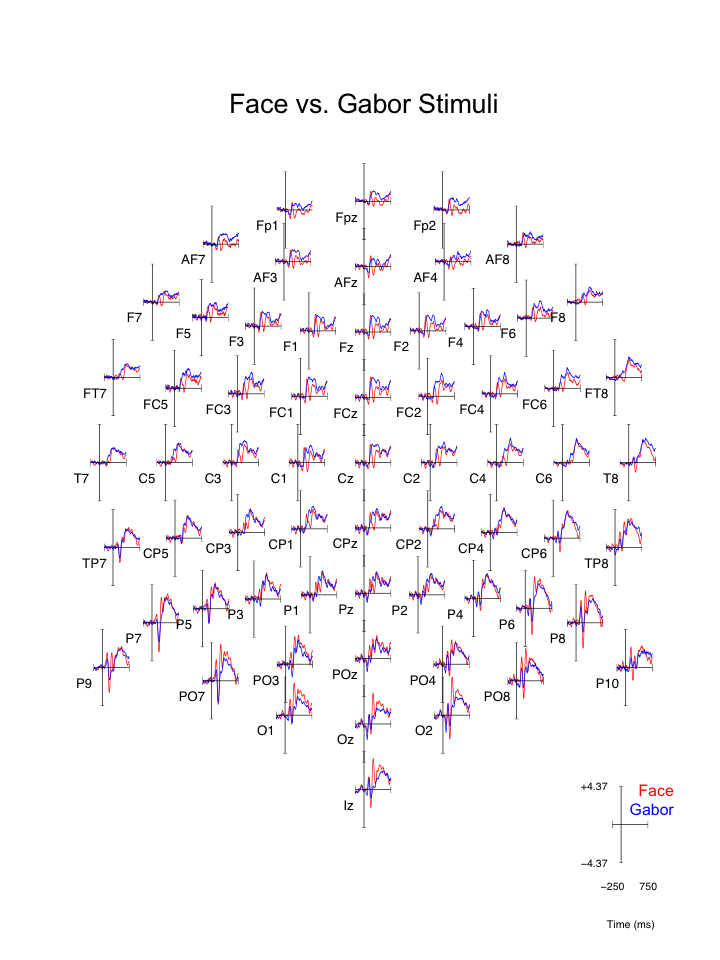

Supplement: S2 Fig — (TIFF) [file pone.0176349.s002.tiff]

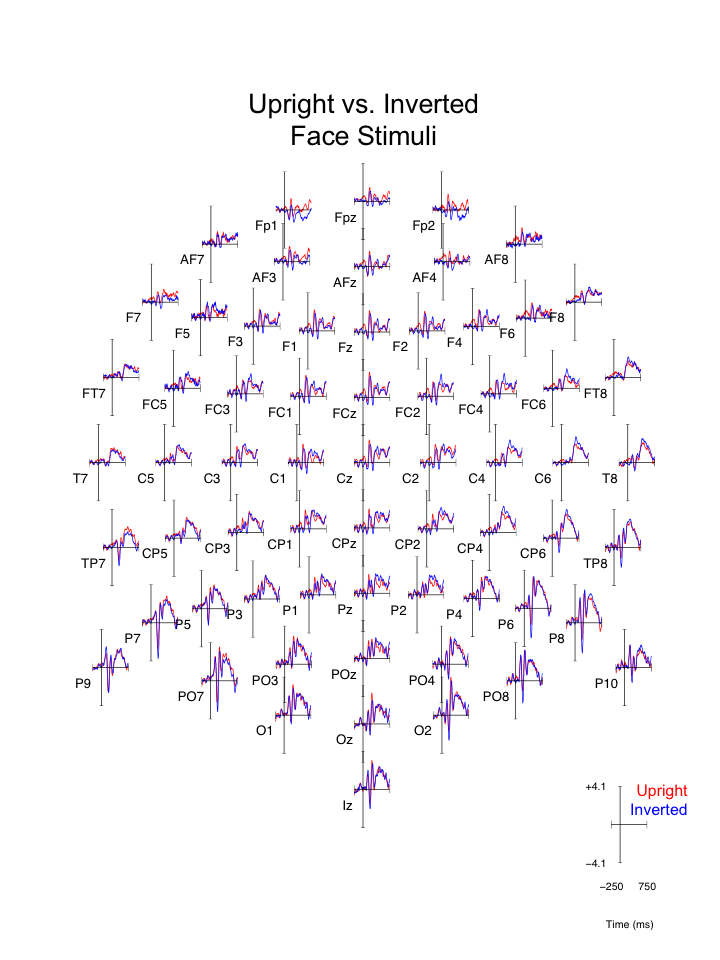

Supplement: S3 Fig — (TIFF) [file pone.0176349.s003.tiff]

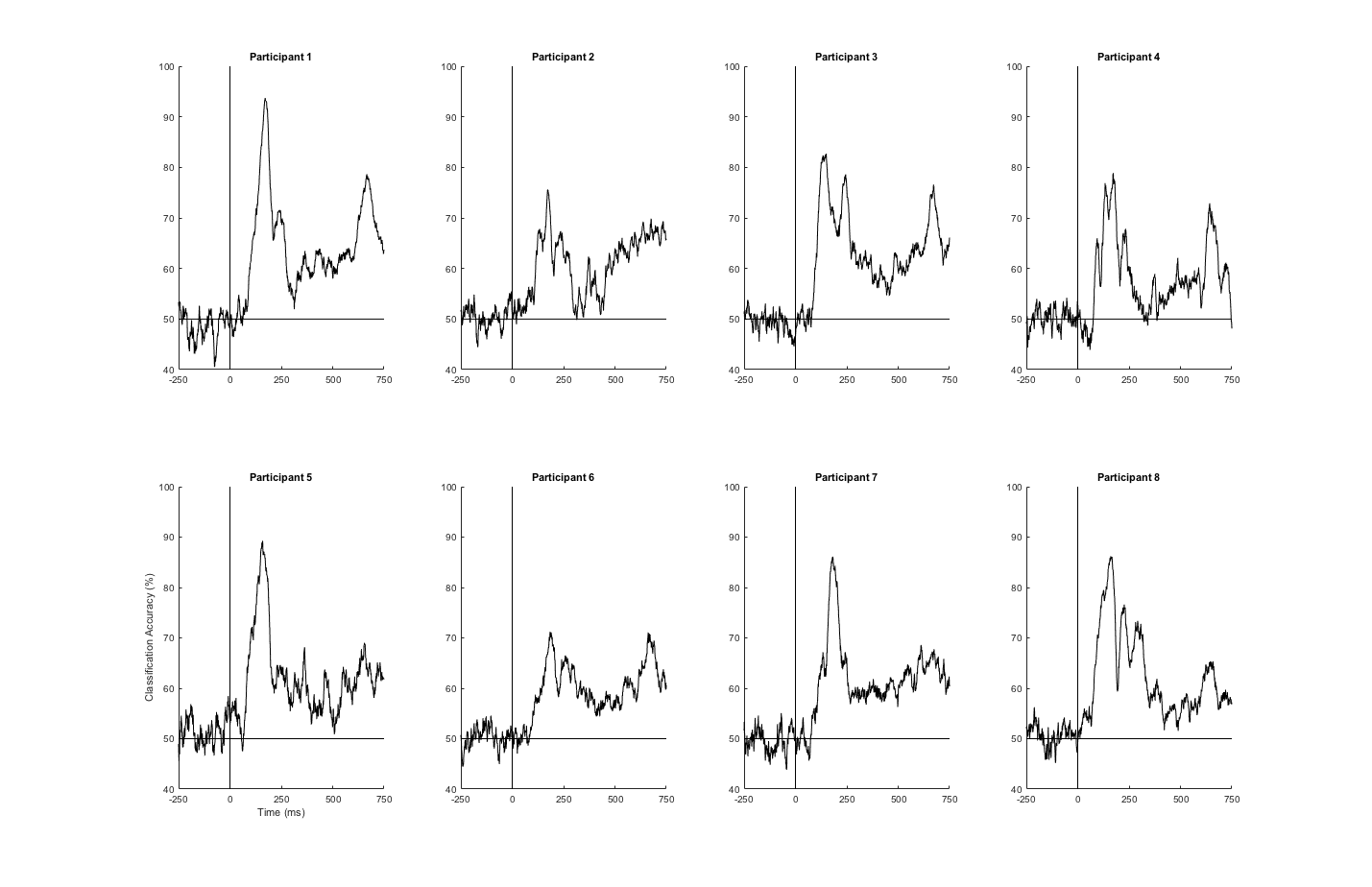

Supplement: S4 Fig — Note that participant 1 was a trained observer. Participant 1 and 4 also participated in Experiment 2. (TIF) [file pone.0176349.s004.tif]

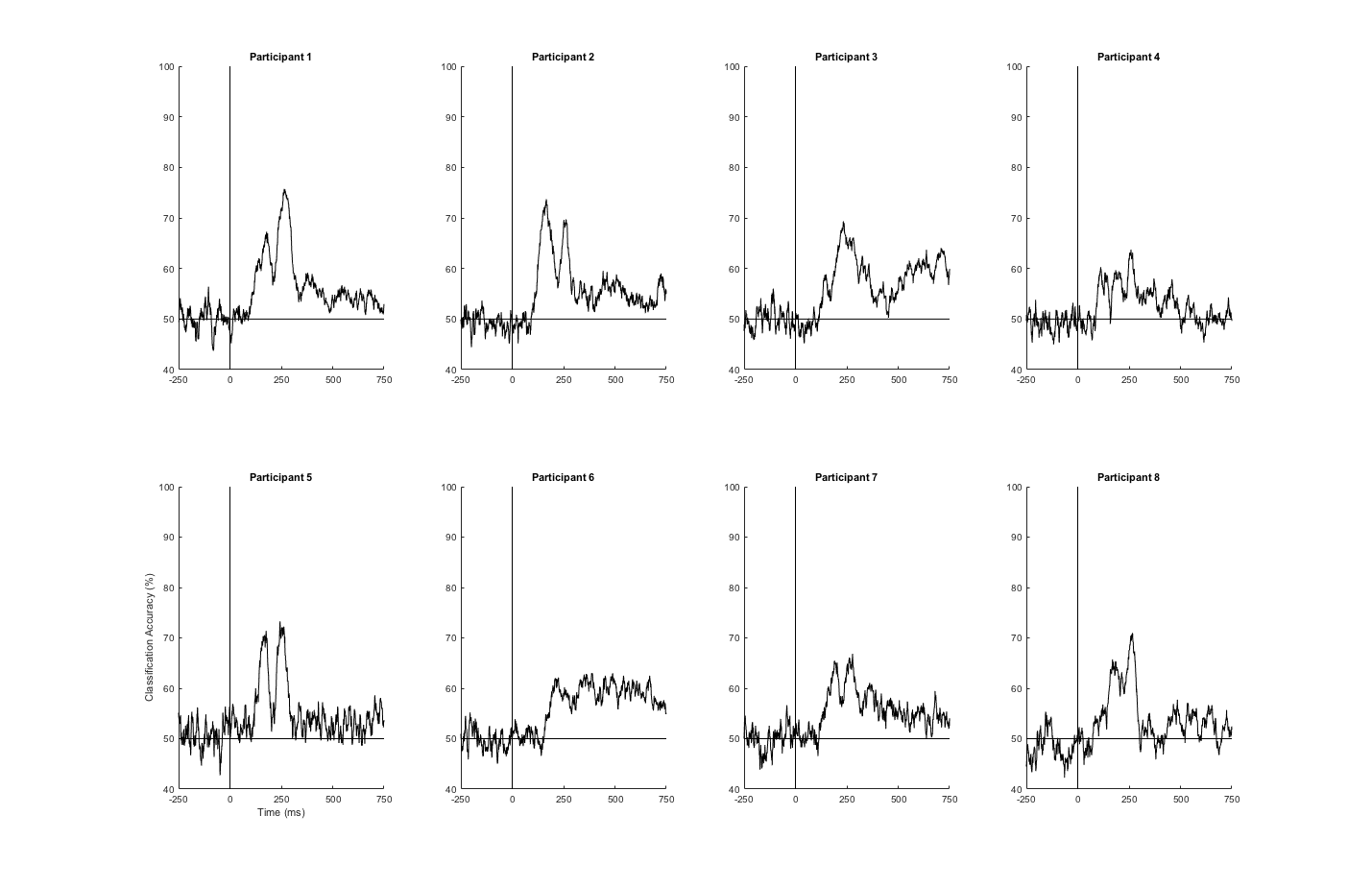

Supplement: S5 Fig — Note that participant 1 was a trained observer. Participant 1 and 4 also participated in Experiment 2. (TIF) [file pone.0176349.s005.tif]

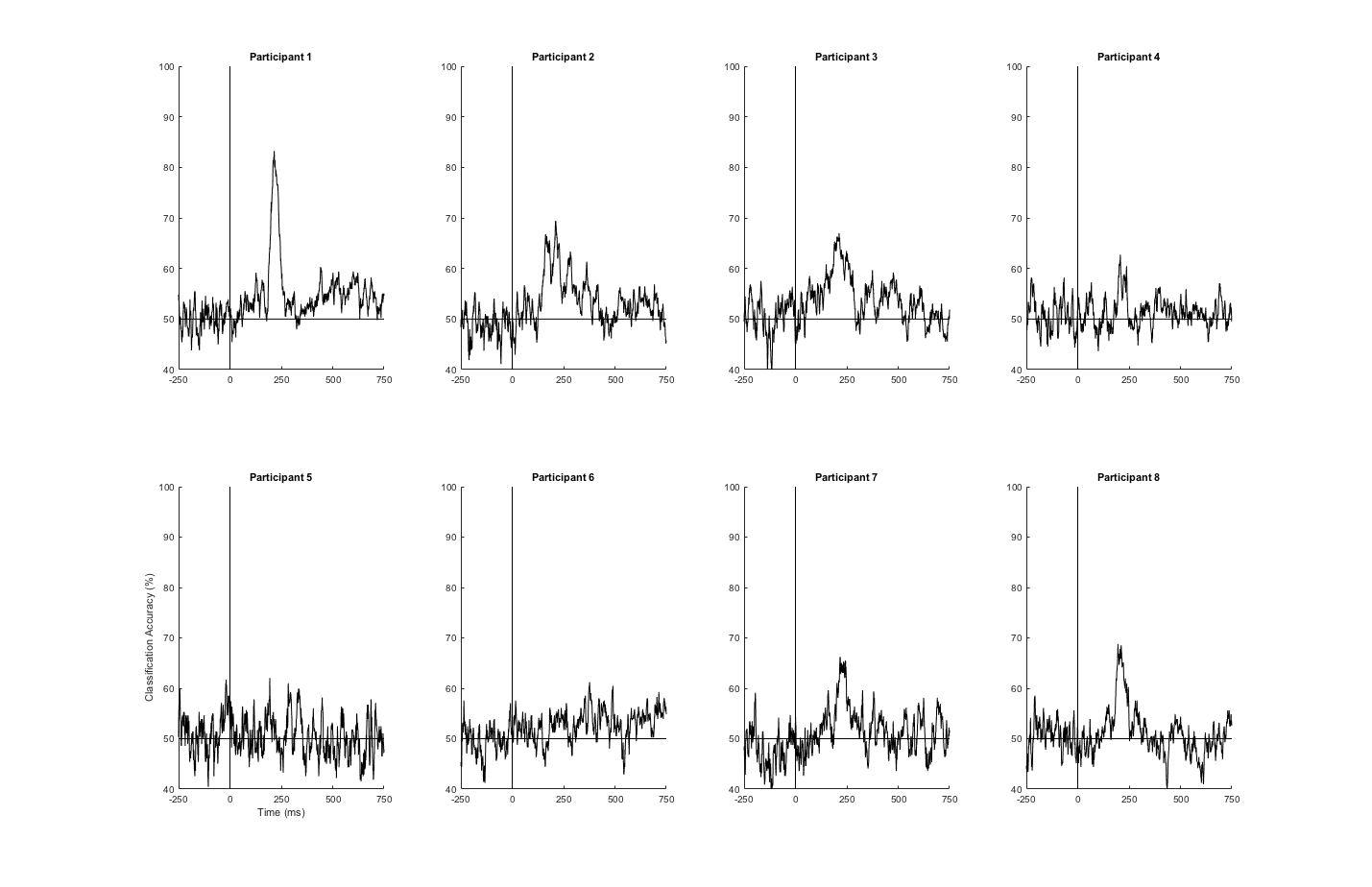

Supplement: S6 Fig — Note that participant 1 was a trained observer. Participant 1 and 4 so participated in Experiment 2. (TIF) [file pone.0176349.s006.tif]

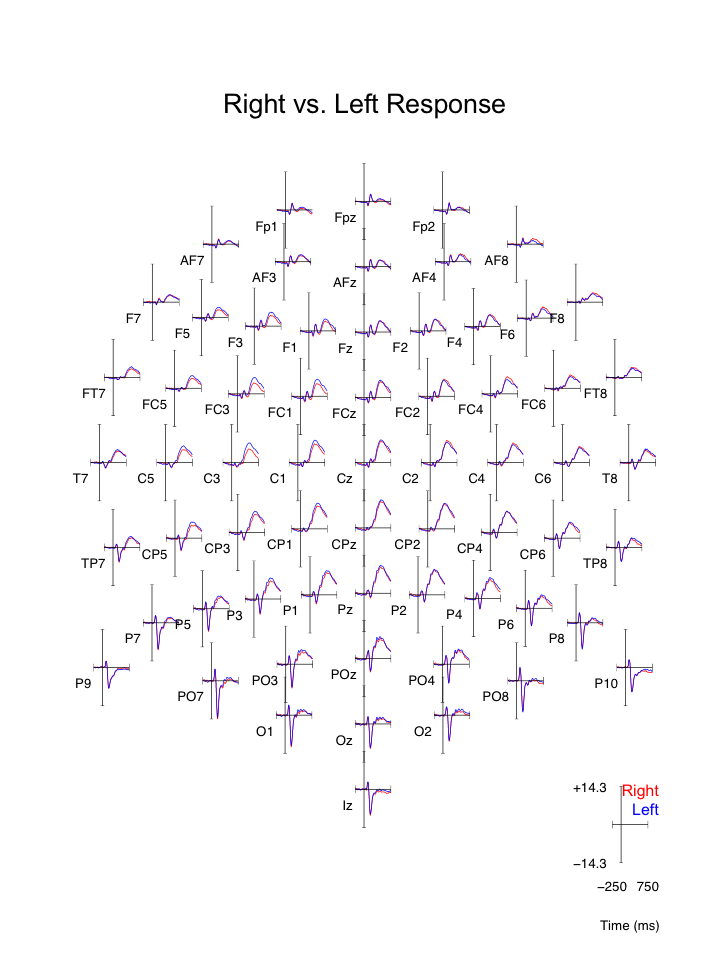

Supplement: S7 Fig — (TIFF) [file pone.0176349.s007.tiff]

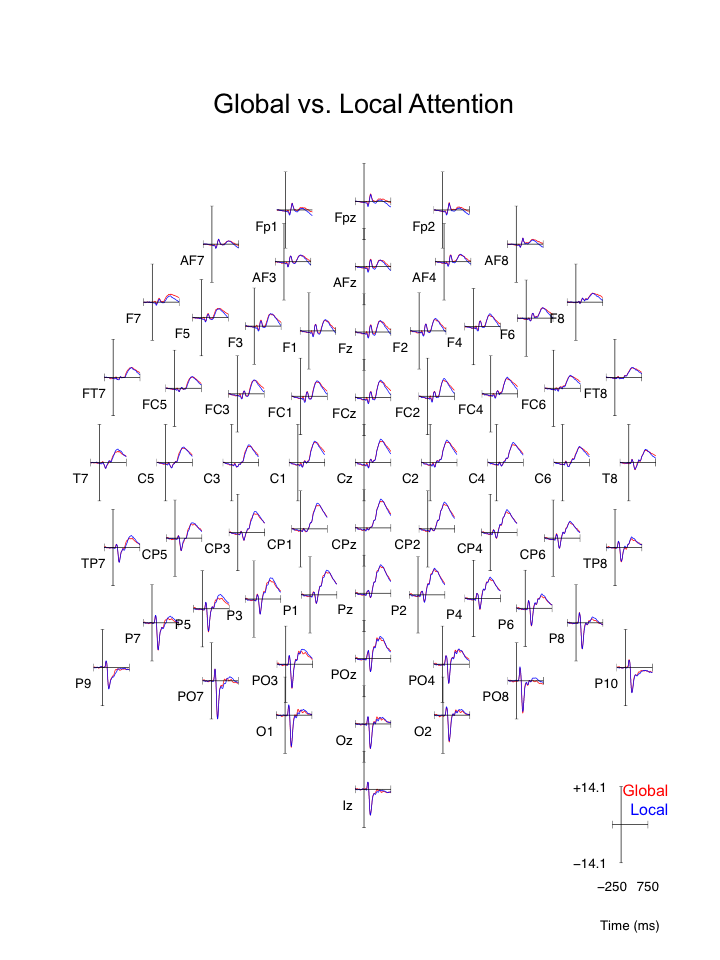

Supplement: S8 Fig — (TIFF) [file pone.0176349.s008.tiff]
